# Perineural invasion predicts poor survival and cervical lymph node metastasis in oral squamous cell carcinoma

**DOI:** 10.4317/medoral.25916

**Published:** 2023-06-18

**Authors:** René Martínez-Flores, Barbara Gómez-Soto, Carlo Lozano-Burgos, Sven Eric Niklander, Márcio Ajudarte Lopes, Wilfredo Alejandro González-Arriagada

**Affiliations:** 1 Unidad de Patología y Medicina Oral, Facultad de Odontología, Universidad Andres Bello, Viña del Mar, Chile; 2 Oral Diagnosis Department, Piracicaba Dental School, University of Campinas, Piracicaba, Brazil; 3 Unidad de Apoyo de Anatomía Patológica, Hospital Carlos Van Buren, Valparaíso, Chile; 4 Unidad de Cirugía Maxilofacial y Odontología, Hospital Carlos Van Buren, Valparaíso, Chile; 5 Facultad de Odontología. Universidad de Los Andes, Chile; 6 Centro de Investigación e Innovación Biomédica, Grupo de Investigación en Oncología Oral. Universidad de Los Andes, Chile

## Abstract

**Background:**

Oral squamous cell carcinoma (OSCC) usually invades peripheral nerves through a process known as perineural invasion (PNI), recognized as an adverse factor considered for the administration of postoperative adjuvant therapy. The aim of this study was to assess the impact of PNI on survival and cervical lymph node metastasis in a cohort of OSCC patients.

**Material and Methods:**

Presence, location and extension of PNI were assessed in a cohort of 57 paraffin-embedded OSCC resections. Clinico-pathological variables were obtained from each case. Five-year overall survival (OS) and 5-year disease-specific survival (DSS) curves were constructed according to the Kaplan-Meier method and compared with log-rank test. The Cox proportional hazard model was used to assess the role of PNI as an independent risk factor related to poor survival, and a binary logistic regression was performed to estimate the predictive value of PNI for regional lymph node metastasis.

**Results:**

PNI was observed in 49.1% of the cases, affecting only small nerves. Peritumoral PNI was the most common location, and multifocal PNI the most frequent extent. Most PNI positive cases had cervical metastasis (*p*=0.001), and PNI was more frequent in stages III-IV than in I-II (*p*=0.02). The five-year OS and the 5-year DSS decreased in PNI positive and peritumoral PNI cases. PNI was an independent risk factor for poor 5-year OS and poor 5-year DSS. The odds for cervical lymph node metastasis were of 6.076 (*p*=0.006) and 10.257 (*p*=0.007) for PNI and Tumor budding (TB) positive cases, respectively.

**Conclusions:**

PNI is a frequent finding in OSCC and an independent risk factor for poor OS and DSS. PNI and TB are both risk factors associated to an increased likelihood for the development of lymph node metastasis. Therefore, we suggest further investigations to test the combined PNI-TB scoring system in risk stratification models for OSCC.

** Key words:**Perineural invasion, tumor budding, oral squamous cell carcinoma, survival, linfonodal metastasis.

## Introduction

Oral cancer is one of the most frequent cancers worldwide. According to GLOBOCAN, 377.713 new cases and 177.757 deaths were registered only in 2020. Oral squamous cell carcinoma (OSCC) is the most common type of oral cancer, associated mainly to chronic consumption of tobacco, alcohol and betel nut, depending on the geographical zone (1).

The understanding of its etiopathogenesis has allowed the development of new and promising treatment alternatives. Surgical approach is the most common form of treatment, which can be accompanied by radio and/or chemotherapy (2). Therapy selection is mainly based on the TNM classification system. However, despite having the same treatment plan, patients within the same TNM stage can have different oncological outcomes. Thus, different authors have explored the use of histological adverse features to complement the TNM staging system, in order to build more precise risk models to aid in the prediction of clinical outcomes with prognostic utility. In this context, the worst pattern of invasion (WPOI), tumor budding (TB), lymphovascular invasion and perineural invasion (PNI) (3-7), have been reported as promising histopathological features to improve the prognostic utility of the TNM classification.

OSCC is considered a malignant neurotropic neoplasm that can involve peripheral nerves of the head and neck region (8). Histopathologically, neural involvement is usually assessed dichotomously (present or absent). Nevertheless, this approach seems to be insufficient (5). Thus, some authors have proposed to further subcategorize neural involvement to improve its ability to predict prognosis (4,9,10). Previous studies in early and advance OSCC cases supports the relationship between PNI and lymph node metastasis, locoregional recurrence and poor overall, disease-free and disease-specific survival (8,10-16), suggesting that PNI is an histopathological feature that should be considered for treatment planning (2). Reports about the prognostic value of PNI are increasing, but its utility in prediction models when combined with other histopathological factors have been poorly explored (17). In this current study, we aimed to assess the utility of PNI, alone or combined with other histological risk factors, for risk estimation of lymph node metastasis, overall survival and other clinical outcomes, in a cohort of OSCC patients.

## Material and Methods

- Study design and sample collection

The cohort included in this retrospective study consisted in 57 consecutive patients diagnosed with primary OSCC. Formalin-fixed paraffin-embedded (FFPE) tissue samples of surgical resections were retrieved from the Pathological Anatomy Unit of Carlos Van Buren Hospital, Valparaíso, Chile.

Only surgical resections of previously untreated tumors were included. Cases without a complete clinical history or with insufficient tissue on the FFPE block were excluded. The following information were extracted from the clinical records: patient sex, age at diagnosis, history of alcohol and/or tobacco consumption, tumor location, TNM classification according to the AJCC 8th edition, treatment (surgery alone or with radiotherapy and/or adjuvant chemotherapy), date of surgery, locoregional recurrence, date of recurrence and, date and cause of death.

- Histomorphological analysis

The histological slides were evaluated and cases were categorized according to the degree of tumor differentiation, WPOI, TB, status of resection margins and the presence of lymphovascular invasion and/or PNI. Tumor differentiation was classified in: well, moderately, or poorly differentiated (7). The WPOI was defined as follows: WPOI 1, broad pushing tumor front; WPOI 2, fingerlike pushing invasion; WPOI 3, large tumor islands > 15 cells; WPOI 4, small tumor island ≤ 15 cells or tumor strands; and WPOI 5, satellite tumor nodule at least 1 mm away from the tumor invasive front (7). TB was defined as the presence of single cancer cells or cluster of less than five cancer cells (3). The status of resection margins margin status was categorized as positive when tumor cells were observed at the tumor margin or negative when tumor margin was free of tumor cells (7). Vascular invasion was defined as tumor clearly within a vascular space (lymphatic or blood vessel) (18). PNI was evaluated with conventional H&E stain, and immunohistochemistry with AE1/AE3 and S100. PNI was considered positive when tumor cells were observed within any of the three layers of the nerve sheath and/or were close to the nerve surroundings in more than a third of its circumference (19). The extent of PNI was reported as unifocal or multifocal when a single or multiple foci of PNI were observed, respectively (10). For each focus of PNI, the distance to the tumor edge was measured. The distance was measured in mm and the tumor edge was considered as 0 mm. Hence, a negative value indicated an intratumoral location, and a positive value indicated an extratumoral location. Moreover, a peripheral location was arbitrarily defined as being −0.2 to 0 mm from the tumor edge. The size of the largest nerve involved was classified as major or minor, depending if the diameter of the nerve bundle was greater or less than 1mm, respectively (9). All histological slides were analyzed by an experienced specialist in Oral and Maxillofacial Pathology (R.M-F).

- Immunohistochemistry

FFPE tissue sections of 3 μm were deparaffinized in xylene and rehydrated in decreasing concentrations of ethanol and distilled water. Antigen retrieval was performed using ImmunoDNA Retriever with Citrate (Bio SB, USA) in a pressure cooker at 95 ºC for 30 minutes, followed by endogenous enzyme blockade using EnVision FLEX peroxidase-blocking reagent (Agilent-Dako, USA) for 10 minutes. The slides were then washed with buffer solution and blocked for 1 hour with 5% serum goat, 4% BSA and 0.1% Triton X-100 in PBS. After that, the tissue sections were incubated overnight with primary antibodies for AE1/AE3 (mouse, monoclonal antibody #53-9003-82, dilution 1:500 Invitrogen Inc., USA) and S100 (mouse, monoclonal antibody 4C4.9, ready to use, Cell Marque, Sigma-Aldrich). The next day, sections were incubated for 30 minutes with a secondary antibody using EnVision FLEX/HRP (Agilent-Dako, USA) and then revelated using diaminobenzidine (DAB) chromogen for 5 minutes. Finally, the slides were counterstained with Harris hematoxylin. Appendix tissue sections were used as external positive controls, and negative controls were obtained by the omission of primary antibodies.

- Statistical analysis

Collected data were summarized through crosstabs, and association between variables was performed using the chi-square test or Fisher's exact test. Survival analysis considered 5-year overall survival (OS), 5-year disease-specific survival (DSS) and 5-year disease-free survival (DFS). OS was defined as the time between the date of primary surgery and the date of death due to any cause. DSS was defined as the time between the date of primary surgery and the date of death due to OSCC. DFS was defined as the time between the date of primary surgery and the date of confirmation of disease recurrence. Survival curves were constructed according to the Kaplan-Meier method and were compared with the log-rank test. To identify independent risk factors related to poor survival, the Cox proportional hazard model was used. To identify risk factors for the development of cervical lymph node metastasis, a binary logistic regression was used. Backward elimination was used to select the covariates included in the regression models. Data were processed using the IBM SPSS Statistics™ software (Version 23) and the level of statistical significance was set at 5% (*P* ≤ 0.05).

## Results

- Clinical findings

Of the 57 cases, 29 were males and 28 females. The age ranged from 28 to 88 years with an average of 62.8 13.8 years. A similar distribution between smokers (49.1%) and non-smokers (50.9%) was observed. Regarding to alcohol consumption, a slight predominance of non-drinkers (57.9%) over drinkers (42.1%) was noticed. Most cases were located on the tongue (57.9%), followed by the alveolar ridge (12.3%) and floor of the mouth (8.8%). Additionally, 56.1% of the cases were classified as T1-T2 and 43.9% as T3-T4. Thirty-three cases did not present lymph node metastasis (57.1%) and there were no cases with distant metastasis. Regarding to the TNM classification, 40.4% of cases were classified as stages I-II, while 59.6% were staged as III-IV (Table 1).

**Table 1:**
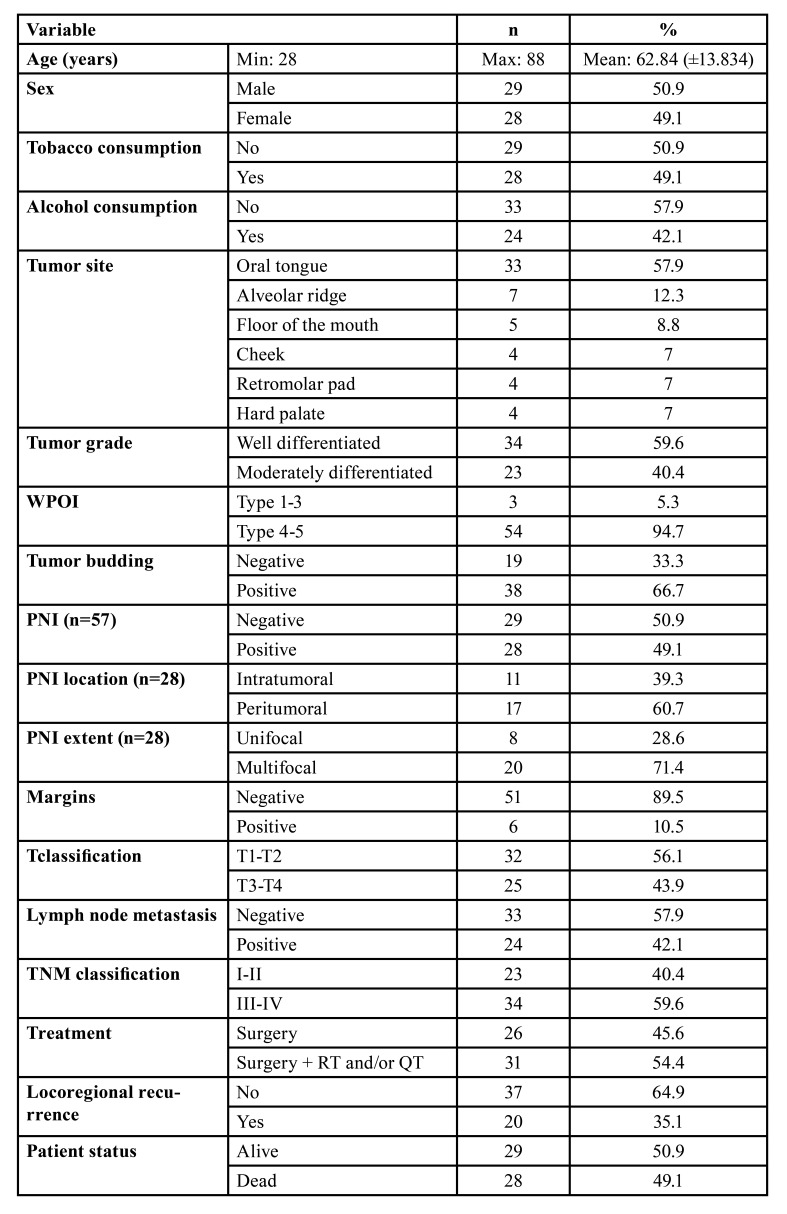
Clinical and histopathological findings of 57 OSCC cases.

- Histopathological findings

Most of the tumors were well differentiated (59.6%), following by moderately differentiated (40.4%). No poorly differentiated cases were observed. WPOI 4-5 was observed in 54 (94.7%) cases and TB in 38 (66.7%) cases.

**Figure 1:**
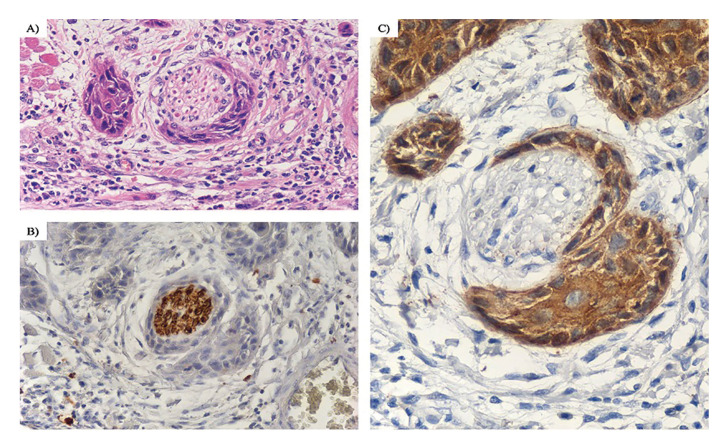
Perineural invasion (40X). Tumor cells are close to the nerve, surrounding more than a third of its circumference. (A) Hematoxylin-Eosin. (B) Invaded peripheral nerve highlighted using S100 immunohistochemistry. (C) Tumor cells are highlighted with AE1/AE3 immunohistochemistry.

PNI was observed in 49.1% of cases, and only small nerves were affected (Fig. 1). Peritumoral PNI was the commonest location (60.7%), followed by intratumoral PNI (39.3%). Regarding to the extent of PNI, multifocal PNI was observed in 71.4% of cases, whereas unifocal PNI was observed in 28.6% of cases (Table 1).

- Association of PNI with clinical and histopathological findings

Presence of PNI was significantly associated with tumor grade (*p*=0.046), being PNI more frequent in moderately differentiated tumors and most PNI positive cases had cervical lymph node metastasis (*p*=0.001). Moreover, PNI was more frequent in stages III-IV than in I-II (*p*=0.02). There were more cases of peritumoral PNI in T3-T4 than T1-T2 tumors (*p*=0.025), as well as a higher number of peritumoral PNI cases with lymph node metastasis (*p*=0.02). In addition, peritumoral PNI was related to III-IV stages (*p*=0.007) (Table 2). There was no statistically significant association between PNI extent and other histopathological variables.

- Survival analysis

Follow-up period was available for all patients of the study cohort. Minimum follow-up period reported was 4 months and the maximum was 200 months. Mean follow-up period was 40,95 ± 41,37 months and a median of 28 months. There were not patients lost. The 5-year OS rate was of 54.4%, which was significantly decreased in PNI-positive tumors (*p*=0.026), PNI with peritumoral location (*p*=0.030), cases with cervical lymph node metastasis (*p*=0.002), cases in stages III-IV (*p*=0.02) and locoregional recurrence (*p*=0.001) (Table 3). The 5-year DDS rate was of 63.2%, which was significantly decreased in PNI-positive tumors (*p*=0.039), PNI with peritumoral location (*p*=0.050), T3-T4 tumors (*p*=0.005), cases with cervical lymph node metastasis (*p*=0.002), tumors in stages III-IV (*p*=0.006) and locoregional recurrence (*p*=0.001) (Table 3). The 5-year DFS rate was of 64.9% and was not significantly affected by any clinical or histopathological variable.

- Clinicopathological prognostic factors

In the Cox univariate analysis, PNI (*p*=0.034), peritumoral PNI (*p*=0.013), cervical lymph node metastasis (*p*=0.004), III-IV stages (*p*=0.027) and locoregional recurrence (*p*=0.002) were risk factors for low 5-year OS. PNI (*p*=0.048), peritumoral PNI (*p*=0.021), T3-T4 tumors (*p*=008), cervical lymph node metastasis (*p*=0.004), III-IV stages (*p*=0.012) and locoregional recurrence (*p*=0.003) were found risk determinants for low 5-year DSS. No risk factors for 5-year DFS were identified on the univariate analysis (Table 4).

**Table 2:**
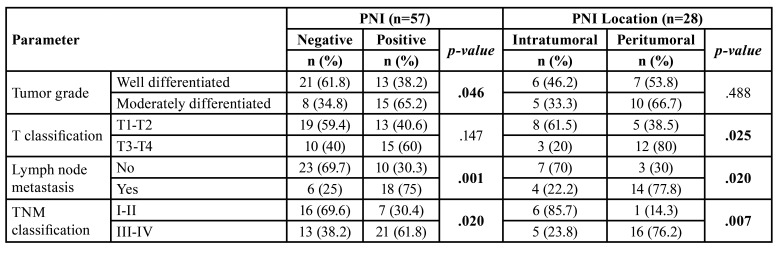
Association of clinical and histopathological findings with PNI.

**Table 3:**
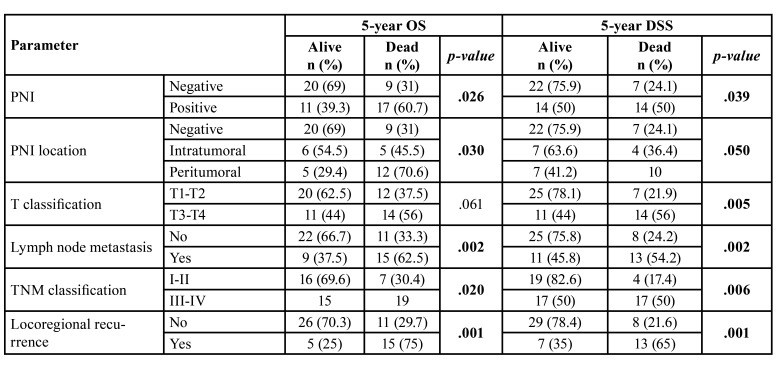
Association of clinical and histopathological findings with 5-year OS and 5-year DSS.

**Table 4:**
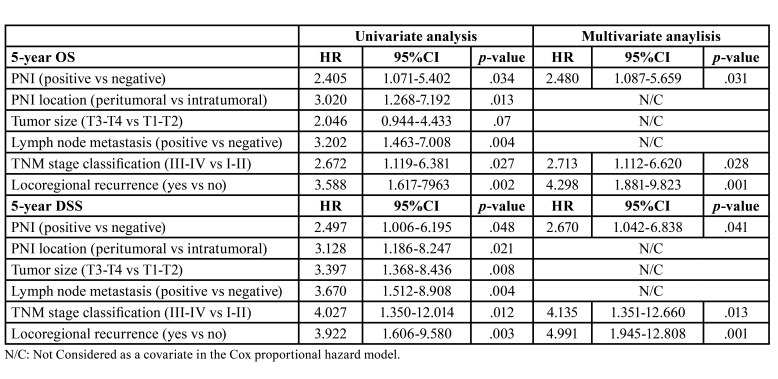
Cox proportional hazard model.

A Cox multivariate regression analysis including only PNI, TNM stage and locoregional recurrence as covariates was performed to detect independent risk factors for 5-year OS and 5-year DSS. We found that PNI (*p*=0.031), III-IV stages (*p*=0.028) and locoregional recurrence (*p*=0.001) were independent risk factors for a poor 5-year OS. Similarly, PNI (*p*=0.041), III-IV stages (*p*= 0.013) and locoregional recurrence (*p*=0.001) were also independent risk factors for a poor 5-year DSS (Table 4).

- Lymph node metastasis prediction model

**Figure 2:**
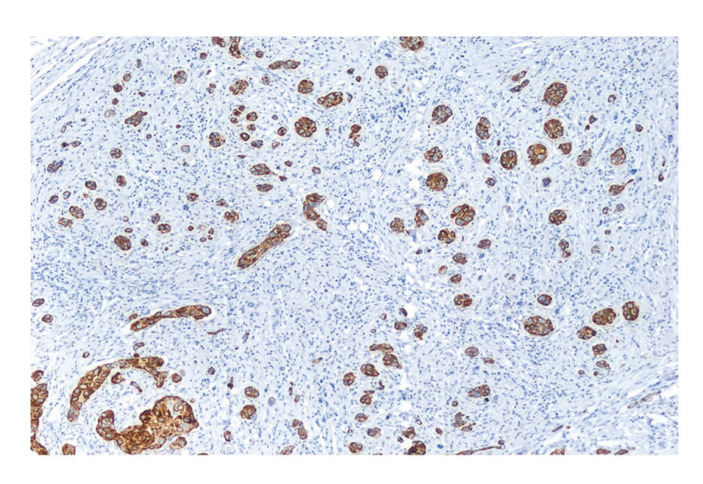
Tumor budding (40X). Single epithelial cancer cells and clusters of less than five epithelial cells at the invasive front. Tumor cells are highlighted with AE1/AE3 immunohistochemistry.

A binary logistic regression was carried out to assess the effect of PNI and others histological features for the development of lymph node metastasis. The odds for cervical lymph node metastasis was of 6.076 (95% C.I: 1.659-22.251, *p*=0.006) for PNI positive cases and of 10.257 (95% C.I: 1.895-55.519, *p*=0.007) for TB positive cases. These data shows that the risk for cervical lymph node metastasis increases 6 to 10 times in cases with PNI and TB (Fig. 2).

## Discussion

OSCC is the most common cancer of the head and neck region. It has traditionally been described as a disease that affects the tongue of smokers and elderly males, however, its sociodemographic profile is changing. A database study evaluating the incidence of cancer in the United States from 2001 to 2017 shows that the male-female ratio has decreased across all age ranges, likely due to the increase of tobacco use in females. Although the tongue continues to be the most common subsite, followed by the floor of the mouth, an increase in the incidence of other subsites such as the gingiva and the palate has been reported (20). These changes in the epidemiology of OSCCC are reflected in our cohort. We found similar number of cases between males and females, smokers and non-smokers, as well as similar distribution by subsites, being the tongue the most common location, followed by the alveolar ridge or gingiva.

OSCC is considered a malignant neurotropic neoplasm that can involve peripheral nerves of the head and neck region (8). In the current cohort, PNI was detected in 49% of the cases (*n*=28), similar to previous reports where the presence of PNI ranged between 17.4% and 50% of all OSCC cases (11,21). In our study the most common PNI location was peritumoral, followed by intratumoral nerve invasion. This differs with previous reports where intratumoral PNI was the most common location, with only 25% of the cases exhibited extratumoral PNI (5). These differences could be explained because in our study, to accurately characterize PNI, we used immunohistochemistry against AE1/AE3 and S100. Immunohistochemistry is a recommended ancillary technique for the diagnosis of PNI in OSCC, given its ability to determine the specific location of the nerve fibers (18), especially in infiltrating tumors with a non-cohesive invasive front, stromal desmoplasia, or an intense inflammatory host response. PNI extent has been classified into unifocal or multifocal, based on the number of nerve fibers affected (10). In our cohort, most cases with PNI had multifocal invasion, similar to other reports (4,10).

Regarding the association between PNI and other variables, we observed that most PNI-positive cases corresponded to moderately differentiated tumors, suggesting that the loss of differentiation could stimulate neural invasion (22). We also detected an association between PNI and cervical lymph node metastasis, which has also been reported by others (11-13,23). Statistically significant associations between PNI location with tumor size and cervical lymph node metastasis were also observed, which is in agreement with previous reports (4,10,12,13,15,21). One of the proposed explanations for this relationship, is that as tumor thickness increases, the invasion of lymph vessels and nerve fibers located in the submucosa is facilitated (24). PNI results from nerve-tumor crosstalk, a complex biological interaction that promotes neurogenesis, tumor proliferation and invasion, and both PNI and lymphovascular invasion share the activation of similar signaling pathways, where neurotropic factors, chemokines and other biomolecules are secreted towards the perineural niche and the tumor microenvironment (25,26). Therefore, when an OSCC has infiltrated nerve fibers, regardless of their location and size, it is likely that lymphatic invasion has also ocurred (16,25,26).

Several studies have reported the role of PNI as independent predictor and its impact on survival in OSCC (Supplement 1). Similar to other studies, we found lower 5-year OS rates in patients with PNI (13,23), specifically associated with peritumoral PNI. It is difficult to compare this with other studies as location of PNI and overall survival is not commonly evaluated. It has been proposed that PNI does not have an impact on DSS (5), however, we observed lower 5-year DSS in cases with PNI. About PNI location and 5-year DSS, our data showed lower survival rates in patients with peritumoral PNI, which differs with a recent study that showed no differences in terms of DSS survival when stratifying PNI by location (5). Although previous studies have associated 5-year OS and 5-year DSS with the extension of PNI, we did not observe that association (4).

To evaluate risk factors related to 5-year survival, we performed a Cox regression model. The univariate analysis showed that PNI and peritumoral PNI location were risk factor associated to a decreased 5-year OS and 5-year DSS. However, when the model was adjusted for TNM classification and locoregional recurrence covariates, only the presence of PNI was an independent risk factor for poor 5-year OS and poor 5-year DSS, which is consistent with observations made from advanced OSCC (11).

Risk models to predict disease behavior contribute to clinical decision making, such as for example, the decision to perform a neck dissection. Under the dilemma of avoiding overtreating or undertreating the patient, the decision is facilitated when the risk factors related to the development of cervical lymph node metastasis are analyzed (27). Through a binary logistic regression, we showed that PNI and TB were risk factors for the development of cervical lymph node metastasis, being the risk higher in tumors with both histopathological findings. TB has been previously reported to be associated with a high risk of nodal metastasis (7,28), mainly due to its association with epithelial-to-mesenchymal transition process (29). According to our results, despite the tumor has been diagnosed in an early stage and the margins from the resection are free of tumor cells, the presence of PNI and TB suggest a worst prognosis.

Several studies have proposed the need to subcategorize PNI (4,9,10), however, our results show that the dichotomic report of PNI (negative or positive) is a good predictor for adverse oncological outcomes. The data set for the report of carcinomas of the oral cavity published by the International Collaboration on Cancer Reporting, proposed that PNI is an independent risk factor associated with poor overall survival, locoregional recurrence and cervical lymph node metastasis. Therefore, it must be reported in the pathological study of OSCC surgical specimens, because is an element that should be considered for clinical management, staging and prognosis (30).

## Conclusions

PNI is a frequent finding in OSCC and an independent risk factor related to poor OS and DSS. Furthermore, the presence of PNI and TB are both risk factors associated with cervical lymph node metastasis. We suggest further investigations to test the combined PNI-TB scoring system in risk stratification models for OSCC.
